# RETRACTED: Wiesinger et al. An Innovative Tool for Evidence-Based, Personalized Treatment Trials in Mucopolysaccharidosis. *Pharmaceutics* 2023, *15*, 1565

**DOI:** 10.3390/pharmaceutics15112596

**Published:** 2023-11-07

**Authors:** Anna-Maria Wiesinger, Brian Bigger, Roberto Giugliani, Christina Lampe, Maurizio Scarpa, Tobias Moser, Christoph Kampmann, Georg Zimmermann, Florian B. Lagler

**Affiliations:** 1 Institute of Congenital Metabolic Diseases, Paracelsus Medical University, 5020 Salzburg, Austria; 2 European Reference Network for Hereditary Metabolic Diseases, MetabERN, 33100 Udine, Italy; brian.bigger@manchester.ac.uk (B.B.); christina.lampe@paediat.med.uni-giessen.de (C.L.); maurizio.scarpa@metab.ern-net.eu (M.S.); 3 Stem Cell and Neurotherapies, Division of Cell Matrix Biology and Regenerative Medicine, Faculty of Biology, Medicine and Health, University of Manchester, Manchester M13 9PL, UK; 4 Department of Genetics, Medical Genetics Service and Biodiscovery Laboratory, Portal Hospital de Clínicas de Porto Alegre, Universidade Federal do Rio Grande do Sul (UFRGS), Casa dos Raros, Porto Alegre 90610-261, Brazil; rgiugliani@hcpa.edu.br; 5 Department of Child Neurology, Epilepetology and Social Pediatrics, Center of Rare Diseases, University Hospital Giessen/Marburg, 35392 Giessen, Germany; 6 Regional Coordinating Center for Rare Diseases, University Hospital Udine, 33100 Udine, Italy; 7 Department of Neurology, Christian Doppler University Hospital, Paracelsus Medical University, 5020 Salzburg, Austria; t.moser@salk.at; 8 Department of Pediatric Cardiology, University Hospital Mainz, 55131 Mainz, Germany; kampmann@uni-mainz.de; 9 Team Biostatistics and Big Medical Data, IDA Lab Salzburg, Paracelsus Medical University, 5020 Salzburg, Austria; georg.zimmermann@pmu.ac.at; 10 Research and Innovation Management, Paracelsus Medical University, 5020 Salzburg, Austria

The journal retracts the article, An Innovative Tool for Evidence-Based, Personalized Treatment Trials in Mucopolysaccharidosis [1], cited above, with the mutual agreement and support of the authors.

Following publication, concerns were brought to the attention of the publisher regarding the use of unpublished trial data without appropriate permission and potential concerns about the accuracy of the data.

Adhering to our complaints procedure, an investigation was conducted that confirmed a lack of appropriate permission from the authors and the conference organizers (Worldsymposium) in using incomplete trial data of anakira in MPS III NCT04018755, incorrectly presenting a placebo in the trial, using a table of incorrect data in the supplementary information, as well as the preliminary nature of this data meaning they could not be yet fully be relied upon to support the main findings of this study, leading to errors in values and calculations. This article is therefore retracted. 

This retraction was approved by the Editor-in-Chief of the journal Pharmaceutics. 

The authors agree to this retraction.

## References

[1] Wiesinger A.-M., Bigger B., Giugliani R., Lampe C., Scarpa M., Moser T., Kampmann C., Zimmermann G., Lagler F.B. (2023). RETRACTED: An Innovative Tool for Evidence-Based, Personalized Treatment Trials in Mucopolysaccharidosis. Pharmaceutics.

